# Eyes wide shut - unusual two stage repair of pectus excavatum and annuloaortic ectasia in a 37 year old marfan patient: case report

**DOI:** 10.1186/1749-8090-6-64

**Published:** 2011-05-02

**Authors:** Martin TR Grapow, Paula Campelos, Clemente Barriuso, Jaume Mulet

**Affiliations:** 1 Department of Cardiovascular Surgery, Hospital Clínic, University of Barcelona, Barcelona, Spain

## Abstract

We report about a 37 year old male patient with a pectus excavatum. The patient was in NYHA functional class III. After performed computed tomography the symptoms were thought to be related to the severity of chest deformation. A Ravitch-procedure had been accomplished in a district hospital in 2009. The crack of a metal bar led to a reevaluation 2010, in which surprisingly the presence of an annuloaortic ectasia (root 73 × 74 mm) in direct neighborhood of the formerly implanted metal-bars was diagnosed. Echocardiography revealed a severe aortic valve regurgitation, the left ventricle was massively dilated presenting a reduced ejection fraction of 45%. A marfan syndrome was suspected and the patient underwent a valve sparing aortic root replacement (David procedure) in our institution with an uneventful postoperative course. A review of the literature in combination with discussion of our case suggests the application of stronger recommendations towards preoperative cardiovascular assessment in patients with pectus excavatum.

## Background

There are no guidelines concerning the clinical evaluation of patients with isolated pectus excavatum prior surgical repair, but some recommendations do exist [1,2]. Besides radiographic evaluation using a computerized tomographic scan (CT), performance of an ECG, transthoracic or transesophageal echocardiogram, pulmonary function testing and cardio-pulmonary exercise testing are suggested. However, the extent of physical und especially image-guided examinations is generally on discretion of the physician in charge. In our patient important signs have been ignored retrospectively, which finally led to an unusual two-stage repair.

## Case presentation

**Figure 1:**
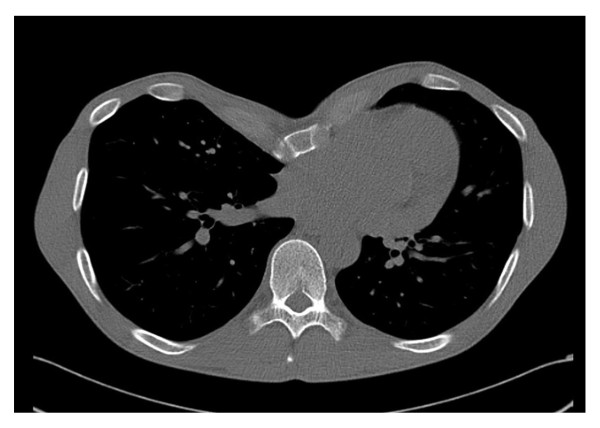
**Initial CT-scan showing the pectus excavatum**.

A 37 year old man was referred to a district hospital with pectus excavatum and progressive shortness of breath. Native computed tomography revealed an excessively deformed chest (Figure 1) and symptoms were thought to be related to the anatomical situation. After presentation of the patient in the thoracic surgery unit, he was scheduled for an operative correction. A Ravitch-procedure had been performed in July 2009. The patient's pectus excavatum was addressed using two metal bars. They were placed in a parallel fashion with the ends supported by the lateral thorax at the level of the third and fourth rib and fixed with ripclamps. The middle portion of the chest was straightened by the bar running underneath it. The patient showed an uneventful post-op course and was discharged on day 9 postoperatively.

**Figure 2:**
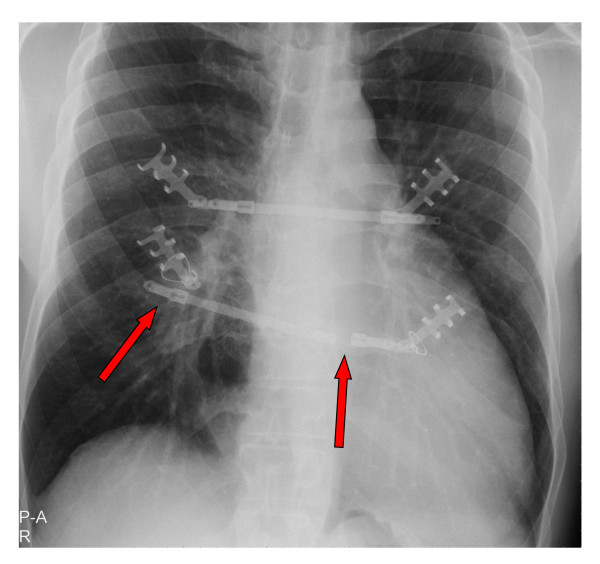
**X-ray at readmission**. Red Arrows indicating the crack and dislocation of the metal bars.

**Figure 3:**
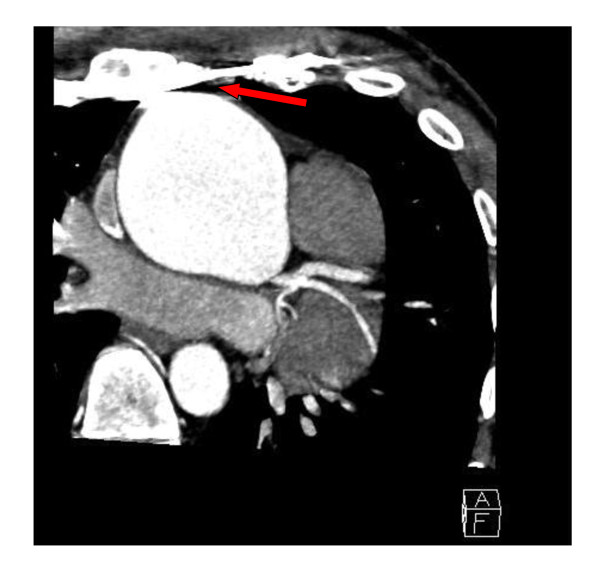
**Follow-up CT-scan**. Red arrow demonstrates the close relationship between one of the metal bars and the annuloaortic ectasia.

Although the anatomical shape was almost normalized after the operative intervention, fatigue, shortness of breath and palpitation were still persistent. A broken and dislocated lower metal bar with concomitant instability (Figure 2) led to a reevaluation in May 2010. After realization of a follow up computed tomography surprisingly and as a co-finding, a severely dilated ascending aorta >7 cm was found, located in direct neighborhood to the metal bars (Figure 3). The following cardiological investigations (echocardiography, MRI) revealed a tricuspid aortic valve with a severe aortic regurgitation due to an annuloaortic ectasia (73 × 74 mm root), a massively dilated left ventricle (LVEDD 85 mm) without hypertrophy and a slightly reduced ejection fraction of 45%. The mitral valve showed a normal morphology with mild regurgitation but with normal annular, valvular and subvalvular conditions.

The patient was transferred to the Hospital Clinic for surgical correction of the cardiovascular pathology. After midline sternotomy the two titan bars were identified. The lower dislocated and broken bar was removed completely, the upper bar was cut and 3 cm were removed. The pericardium was totally intact after the Ravich-procedure. After opening of the pericardium the huge annuloaortic aneurysm became visible. After heparinization and installation of the extracorporal circulation with aortic cannulation of the arch and venous cannulation using a two-stage cannula placed into the right atrium cardiopulmonary bypass was started. The ascending aorta was distally crossclamped directly underneath the brachiocephalic trunc. After inspection of the aortic valve and almost complete resection of the ascending aorta, a valve sparing aortic root replacement (David procedure) using a straight 30 mm Hemashield prosthesis with lateral insertion of the coronary ostia was performed. The echocardiography showed a perfect valve function with low gradients. After weaning from bypass and decannulation protamin was substituted. The sternum was closed using the Robiscek wire reinforcement technique. Apart from a short period of atrial flutter and a spontaneously resolved paralytic ileus the patient's postoperative course was uneventful and he was discharged at day 10 postoperatively.

## Discussion

Historically, when underlying anatomical structures were not considerably affected by the pectus excavatum, a two-stage repair with a first intervention focused on the cardiovascular pathology followed by a second operation addressing the thoracic wall was recommended [3]. Simultaneous repair of both lesions was discouraged because of concerns regarding the potential for major complications, such as limited exposure of the heart, excessive bleeding, and increased risk of wound infection. In the last decade reports about successful simultaneously performed single-stage repairs became evident [4-6].

**Figure 4:**
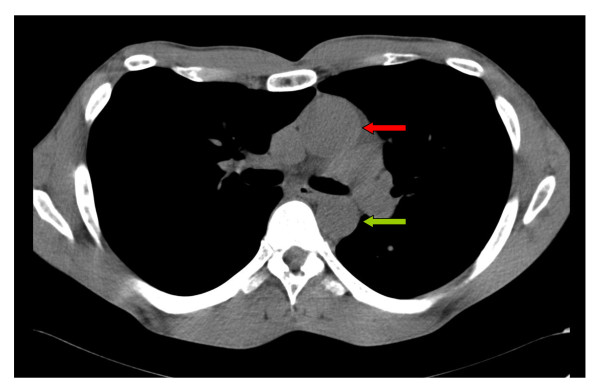
**Initial CT-scan showing the ascending (red arrow) and descending aorta (green arrow)**. The ascending aorta appears to be significantly more dilated as it should be.

We now report about an unusual two-stage repair of a pectus excavatum and an annuloaortic ectasia in a 37 year old marfan patient. Due to a missed finding of an enlarged aorta in the initial computed tomography without contrast medium and the lack of essential diagnostics preoperatively our patient was first operated on the chest deformity using the Ravitch-Procedure. As seen in Figure 3 the bars dangerously almost touch the dilated aorta, which in fact is only covered by the pericardium. Fortunately the early crack in one metal bar combined with partial instability (Figure 2) led to a follow-up examination before a potentially hazardous alteration of the aortic situation may have occurred. The following diagnosis of the annuloaortic dilation points out the importance of a thoroughly raised history and physical examination. Retrospectively, three Marfan criteria could have been detected preoperatively by physical examination in our patient apart from the annuloaortic ectasia, i.e. pectus excavatum, arm span to height ratio >1.05 and facial appearances with dolichocephaly and enophtalmos. Careful assessment of the initial native computed tomography of the chest furthermore could have raised suspicion in an aortic enlargement (Figure 4), but this remains to be difficult to postulate without having the perfect section and additionally lack of contrast medium. Although the association of pectus excavatum with aortic root dilatation is not sufficient to fulfill the Gent criteria for the diagnosis of Marfan syndrome [7], a connective tissue disorder seemed not to be excludable even without the knowledge of the aortic pathology.

Rhee and colleagues [8] report about children with isolated pectus excavatum without a suspected connective tissue disorder, who were referred for routine echocardiographic evaluation. Importantly they found a significantly higher prevalence of aortic root dilatation in those children compared to an age-matched control population.

## Conclusion

In our opinion a careful cardiological assessment in patients with pectus excavatum should be obligatory. Driven by our experience and literature besides CT we strongly recommend to perform screening echocardiography, a non-invasive, safe and inexpensive method, in all patients even with isolated pectus excavatum in order to identify those patients with concomitant cardiovascular manifestations.

## Consent

Written informed consent was obtained from the patient for publication of this case report and any accompanying image. A copy of the written consent is available for review by the Editor-in-Chief of this journal.

## Competing interests

The authors declare that they have no competing interests.

## Authors' contributions

All authors contributed in case management, manuscript preparation and image acquisition. All authors read and approved the final manuscript.
